# Coupling Genetic and Species Distribution Models to Examine the Response of the Hainan Partridge (*Arborophila ardens*) to Late Quaternary Climate

**DOI:** 10.1371/journal.pone.0050286

**Published:** 2012-11-19

**Authors:** Jiang Chang, De Chen, Xinping Ye, Shouhsien Li, Wei Liang, Zhengwang Zhang, Ming Li

**Affiliations:** 1 Key Laboratory of Animal Ecology and Conservation Biology, Institute of Zoology, Chinese Academy of Sciences, Beijing, China; 2 MOE Key Laboratory for Biodiversity Science and Ecological Engineering, College of Life Sciences, Beijing Normal University, Beijing, China; 3 Department of Natural Resources, Faculty of Geo-Information Science and Earth Observation (ITC), University of Twente, Enschede, The Netherlands; 4 Department of Life Science, National Taiwan Normal University, Taipei, China; 5 Ministry of Education Key Laboratory for Tropical Plant and Animal Ecology, College of Life Sciences, Hainan Normal University, Haikou, China; Monash University, Australia

## Abstract

Understanding the historical dynamics of animal species is critical for accurate prediction of their response to climate changes. During the late Quaternary period, Southeast Asia had a larger land area than today due to lower sea levels, and its terrestrial landscape was covered by extensive forests and savanna. To date, however, the distribution fluctuation of vegetation and its impacts on genetic structure and demographic history of local animals during the Last Glacial Maximum (LGM) are still disputed. In addition, the responses of animal species on Hainan Island, located in northern Southeast Asia, to climate changes during the LGM are poorly understood. Here, we combined phylogeographic analysis, paleoclimatic evidence, and species distribution models to examine the response of the flightless Hainan Partridge (*Arborophila ardens*) to climate change. We concluded that *A. ardens* survived through LGM climate changes, and its current distribution on Hainan Island was its *in situ* refuge. Range model results indicated that *A. ardens* once covered a much larger area than its current distribution. Demographic history described a relatively stable pattern during and following the LGM. In addition, weak population genetic structure suggests a role in promoting gene flow between populations with climate-induced elevation shifts. Human activities must be considered in conservation planning due to their impact on fragmented habitats. These first combined data for Hainan Partridge demonstrate the value of paired genetic and SDMs study. More related works that might deepen our understanding of the responses of the species in Southeast Asia to late Quaternary Climate are needed.

## Introduction

Pleistocene climatic changes left strong footprints on the diversity and structure of contemporary northern hemisphere wildlife by ice coverage and caused their distributions to shift [1]–[5]. In addition, it has been proposed that mountains areas in the tropics provide stable moist habitats during ice ages, in which the response of species to climate change can be adjusted with altitudinal shifts, as indicated in southern Europe [3], [6]. There is growing evidence, however, that the largest geographic factor of Southeast Asia, which was not glaciated, was sea level fluctuation during the late Pleistocene [7]. The factor was characterized by a mean annual temperature drop of 4–6°C and drier climate during the Last Glacial Period (LGP) (125–10 kyr ago), which reduced global sea level and exposed the continental shelf throughout Southeast Asia [8]–[11]. The land area consequently varied even twice as much as sea levels fluctuated ±50 m with each of the ∼50 Pleistocene glacial cycles, which resulted in the expansion and contraction of species populations with oscillations in land area and seasonality [7], [12], [13]. However, the impacts of sea level fluctuations on populations and species are still debated [14], [15]. Some studies have indicated that a broad continuous lowland tropical rainforest and savanna over major portions of Sundaland was maintained by lower sea levels during the Last Glacial Maximum (LGM) [7], [14]–[16]. These findings suggest that animal populations extended their distribution to cope with the LGP, particularly the LGM, as supported by pollen analysis of river and offshore sediments from the southern China Sea [17], [18]. Conversely, some genetic studies have shown that migration between Sumatra and Borneo was extremely limited during the LGP despite many major islands being connected for the last 70 kyr, which supports the rainforest refugia hypothesis [19]–[22]. To date, these studies have mainly focused on the evolutionary history of species on Sundaland, Sumatra, and Borneo. The influence of sea level-induced distribution shifts on population genetic structure and demographic history of species from Hainan Island in northernmost Southeast Asia remains poorly understood.

Hainan Island has experienced connection-disconnection events with the Chinese mainland as well as Taiwan Island and Indochina due to sea level fluctuations throughout the late Pleistocene [23], [24]. Therefore, Hainan Island represents a typical land bridge island and can help determine the effects of environment changes on population genetic structure and demographic history for local species, especial flightless animals, during the late Pleistocene ice ages [25]–[28]. Additionally, as Hainan Island is a biodiversity hotspot [29], [30], establishing the phylogeographic patterns of this species may improve management practices for the preservation of endemic wildlife.

More recently, phylogeographic analysis has been integrated with species distribution models (SDMs) as a powerful multi-faceted approach to resolve the processes of how current distribution patterns of genes, populations, and species were shaped [31]. SDMs predict the potential distribution of species based on present occurrence points extrapolated to areas with similarly suitable ecological conditions [32]. Moreover, transferring the current climate envelope of a species onto past climate models gives an estimation of the species’ potential paleodistribution [33]. Therefore, SDMs provide a means of characterizing the spatial distribution of suitable conditions for species and have been widely applied to determine potential distributional areas and refugia during the late Quaternary periods, such as the LGM and present-day [31], [34]–[36]. Specifically, SDMs and phylogeographic analyses are complementary and can provide information about potential dispersal corridors because the inferences from one approach can be explored and potentially validated by the other [35]. SDMs have been applied to explore speciation mechanisms [6], [37], species extinction, niche shifts [38], [39], and increase the realism of historical models to improve phylogeographic inference [31].

Here, we combined an analysis of population genetic structure and demographic history with SDMs to reveal the effect of historical climatic changes on the endemic Hainan Island species (*Arborophila ardens*) and its conservation implications. The Hainan Partridge is mainly restricted in tropical evergreen forest, both broadleaved and mixed coniferous-broadleaved, usually between 600 m and 1,600 m on Hainan Island [40]–[42]. During the past decades, this species' population is suspected to have rapidly declined, in line with habitat loss and degradation within its range. However, the discovery of a number of new populations since 2002 and the subsequent protection of more forest where the species occurs suggests that this rate of decline may have slowed or even ceased in recent years [43], [44].

With multiple segments of mitochondrial DNA (mtDNA) genes and SDMs, the present study aimed to explore the responses of *A. ardens* to the late Quaternary climate changes by determining the patterns of population genetic structure, demographic history and the distribution shifts. Additionally, we aimed to provide base-line information for conservation and management of the vulnerable endemic species. The results will provide a better understanding of the factors that shape the evolutionary history of biological communities at an island scale and its conservation implications.

## Materials and Methods

### Example Collection, DNA Extraction, PCR Amplification and Sequencing

Until now, suitable forest habitats of *A. ardens* are estimated to cover a total of 660 km^2^, of which 410 km^2^ is in natural reserves. Almost all of the extant populations are distributed in Bawangling, Jianfengling, Diaoluoshan, Limushan and Nanweiling Nature Reserves [40], [43]. The recent survey of population size of this species indicated that its total size was about 1200 [45], much smaller than the numbers reported by BirdLife International [44]. In this work, sixty two samples (blood and muscles) were collected throughout three main distribution regions of *A. ardens* on Hainan Island from a period of 2007–2009 (Bawangling, Jianfengling and Yinggeling Nature Reserves). Collection permission was granted by the regional forestry departments, and the sample collection was carried out under the Guide for the Care and Use of Animals of the Institute of Zoology, Chinese Academy of Sciences with the approval of State Forestry Administration of China.

Total DNA was extracted based on the TIANamp blood genomic DNA extraction kit (TIANGEN, China). The whole mitochondrial CYTB and ND2 were amplified with primers CytbL (5′- TCAACCACACTTCACACAGGC -3′), CytbH (5′-GGTTTACAAGACCAATGTTTTTCA -3′) (designed by the authors), and ND2L (5′- TATCGGGCCCATACCCCGAATAT-3′), ND2H (5′-CTTTGAAGGCCTTCGGTTTA -3′) [46] respectively. Polymerase chain reaction (PCR) amplification was performed in 25 µl reaction volumes with 100 ng of genomic DNA, 0.3 µM of each PCR primer and 10 µl Premix *EX Taq* (TaKaRa). The PCR profile was 94°C for 5 min, followed by 35 cycles of 94°C for 40 s, 52°C for 40 s, 72°C for 1 min and 8 min at 72°C. Each round of PCR reactions also included one negative control to check for contamination. The PCR products were sequenced on a 3730 DNA sequencer (Applied BioSystems). Both strands of each PCR product were sequenced. Sequences were compared visually to the original chromatograms to avoid reading errors and were checked with published DNA data. To avoid amplifying mitochondrial DNA homologues from the nuclear genome (numts), we verified the sequences as follows: 1) designed specific primers, which included those amplifying longer segments of mtDNA (>1 kb); 2) checked the presence of PCR ghost bands, or extra bands; 3) detected the presence of sequence ambiguities, frame-shift mutation and stop codon; 4) used BLAST to do sequence similarity search and alignment with the published sequence databases of related species deposited in GenBank; and 5) detected whether unexpected phylogenetic placements existed in a phylogenetic analysis.

### Molecular Data Analysis

The complete ND2 and CYTB sequences were obtained by aligning the partial sequences with the software SeqEdit (Applied Biosystems Inc., USA). The number of segregating sites, haplotype diversity, and nucleotide diversity for each sampling location were estimated using DnaSP 5.0 [47]. A median-joining network based on maximum parsimony was also used to reconstruct the phylogenetic relationships among haplotypes using NETWORK 4.5.1.6 [48]. Several commonly used methods were applied to test the population expansion of *A. ardens*. First, statistical tests designed to assess whether nucleotide polymorphisms deviated from expectations under the neutral theory -Tajima’s D and Fu’s *Fs* test - were carried out in MEGA 5 [49]. We also calculated Fay and Wu’s H value [50] with its sister species *A.brunneopectus* as outgroup. In general, signals of selective sweeps will result in non-zero D and a significant value of H. Thus, combining D, *Fs* and H tests may allow us to distinguish population fluctuations from selection. Significance of D and *Fs* values were determined using 1,000 simulated samples to produce an expected distribution under selective neutrality and population equilibrium. Second, the ARLEQUIN 3 program [51] was used to compare the observed frequency distribution of pairwise nucleotide differences among haplotypes with that expected from a population under expansion (mismatch distribution analysis). The mismatch distribution is usually multimodal in samples drawn from populations at demographic equilibrium. The validity of the model was tested by obtaining the sum of squared differences (SSD) between the observed and estimated mismatch distribution. In addition, the demographic history was assessed using a Bayesian coalescent method implemented in the BEAST 1.6.1 [52]. We selected the model of nucleotide substitution using jModelTest 0.1.1 [53]. A simple HKY nucleotide substitution model, with the strict molecular clock and the relaxed clock under uncorrelated log-normal distribution, was used to avoid over-parameterization due to the limited diversity of our data. The Posterior distributions of parameters used three independent Markov chain Monte Carlo (MCMC) runs of 2x10^9^ steps, with 10% steps burn-in and were displayed using Tracer 1.4 [54]. Analyses were repeated twice using different random seeds to test for convergence. In all analyses, ESS values for all parameters should exceed 200.

### Species Distribution Models

To obtain an independent perspective on the consequences of climate change for range fluctuations in *A. ardens*, a SDM based on current climate data was used. Assuming niche conservatism over time, this model was projected onto climate reconstructions for both the present-day and LGM [55], [56]. To ensure the samples from the current study are representative of its current distribution, we developed a present-day SDM for *A. ardens* based on the largest-ever occurrence sites (from not only the current samples occurrence sites, but also the five historical occurrence records) throughout the Hainan Island based on our previous fieldwork [42], [57]. Specifically, Moran's I statistic showed that spatial autocorrelation was not significant in occurrence samples (Moran’s I = 0.373, Z = 1.49, P>0.05). Then we projected the SDM to LGM conditions across the areas of Southeast Asia. We applied the Maxent algorithm to construct SDM, which is a machine-learning technique based on the principle of maximum entropy that fits a probability distribution to the environmental conditions at the locations where a species has been observed [58]. The 19 bioclimatic variables (Table S1) were downloaded from the WorldClim Dataset (www.worldclim.org) for constructing SDM. These climate layers for present-day are based on spatially interpolated values of temperature and precipitation gathered from weather stations around the world from 1950–2000 [59], whereas the bioclimatic variables for the LGM were generated from the climate reconstructions based on MIROC 3.2 [60]. The default convergence threshold and maximum number of iterations (1000) values were used with 75% of localities for model training. We let the program select suitable regularization values and functions of environmental variables automatically based on sample size. Model performance was evaluated using the Area Under the Curve (AUC) values of the Receiver Operating Characteristic (ROC) and the threshold dependent binomial omission tests calculated by Maxent. The AUC values are expressed as the ratio of the area under the observed curve (i.e. the overall area for which each algorithm predicts as present) to the area under the line that defines a random expectation [61]. The continuous logistic output indicates the relative suitable environmental conditions for the species (based on the principle of maximum entropy, but constrained by the input occurrence data). All inputs and outputs were developed at a spatial resolution of 2.5′.

## Results

### Species Phylogeography

With the combination of the complete 1041 bp ND2 and 1143 bp cyt *b* of mtDNA from 62 *A. ardens* individuals, we obtained ten haplotypes defined by twelve variable sites without stop codons and deletions/insertions (GenBank Accession number: JQ825228-JQ825239). The combined haplotype diversity (*h*) was 0.78 over samples, while it also exhibited an extremely low nucleotide diversity (*π*) ( = 0.062%) (as shown in Table 1).

**Table 1:** Genetic information in four sampling regions of *Arborophila ardens* based on two mtDNA genes (CYTB and ND2).

Sampling location	*N*	Haplotypes	Polymorphic sites	*h*	*π*	Tajima’s D
Nanweiling	30	7	6	0.731	0.00044	−1.069^a^
Bawangling	20	6	8	0.842	0.00097	−0.239^a^
Yinggeling	12	4	3	0.636	0.00034	−0.829^a^
Overall	62	10	12	0.781	0.00062	−1.348^a^

a *P*>0.05.

**Figure 1:**
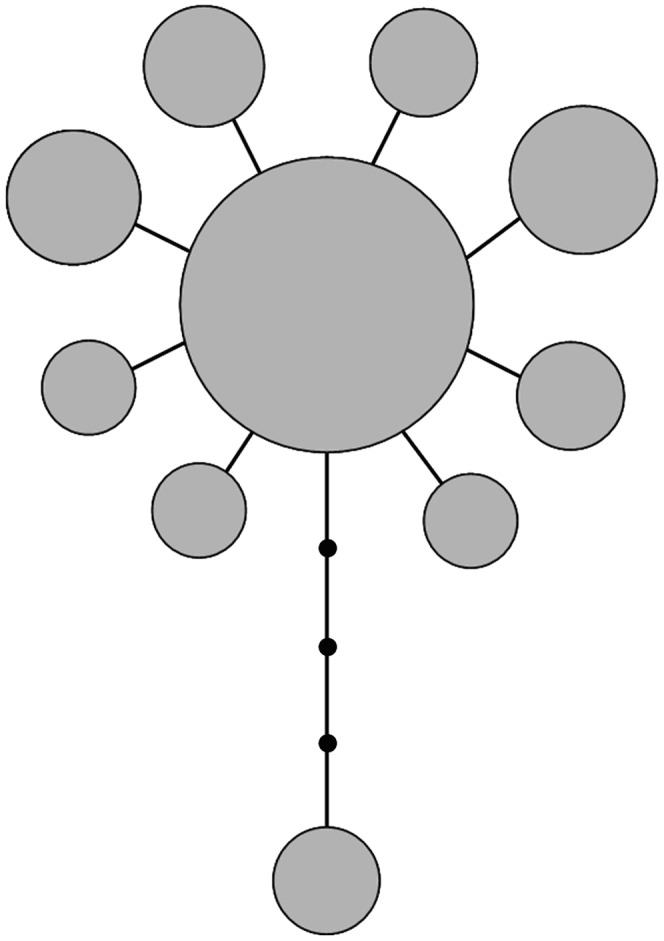
Unrooted haplotype network analysis of *A. ardens* based on mtDNA CYTB and ND2 sequences. Each circle represents a unique haplotype. The size of the circle indicates the number of individuals of each haplotype. Each black dot represents a single mutational change according to length.

**Figure 2:**
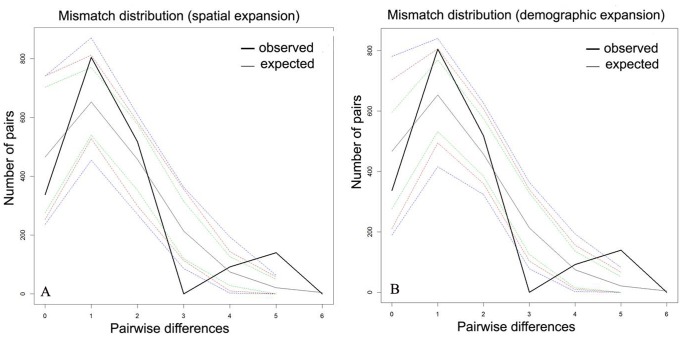
Mismatch distribution of expected and observed with confidence intervals (A: spatial expansion; B: demographic expansion) of *A. ardens*. On the horizontal axis is the number of nucleotide site differences between pairs of individuals.

**Figure 3:**
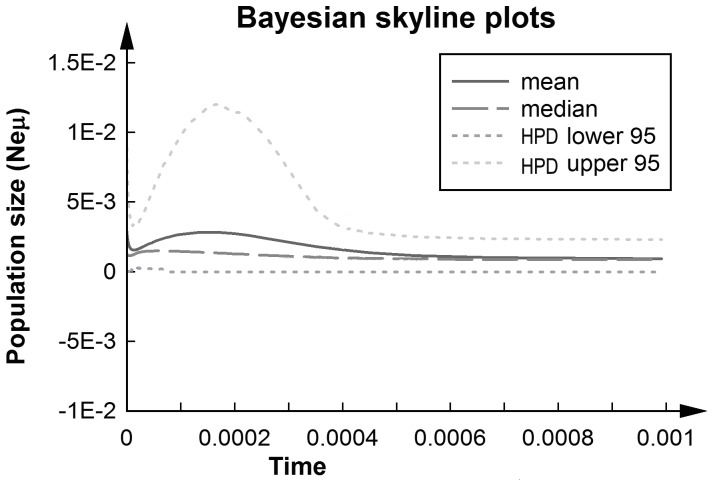
Bayesian skyline plot of effective population size (scaled by mutation rate) through time. Estimates are shown along with the union of the 95% highest posterior density (HPD) areas. Note that the time scale begins with the present on the left and is given in substitutions per site, which can be converted to units of time via a molecular clock calibration.

Minimum spanning network (MSN) parsimony showed a star-like topology, with a dominated haplotype shared between sampling locations and circled by all other rare haplotypes (Fig. 1). The haplotype distribution frequency and network analysis did not reveal the geographic structure of *A. ardens* (F*st*<0.001). Both Fu’s *Fs* and Tajima’s D statistics showed non-significantly negative value, and failed to reject the null hypothesis of neutral evolution and demographic equilibrium of the CYTB and ND2 (Table 1). Likewise, Fay and Wu’s H was not significant (*p* = 0.3), which could be the indicator of rejection of the selective sweep. Moreover, the mismatch distributions were multimodal-shaped curves based on the combined sequences (Fig. 2), with the SSD *P* value <0.05 (0.03 and 0.001 for demographic and spatial expansion, respectively). Tests of sudden population growth based on mismatch distributions statistics could be rejected. In addition, by using the 2% Myr^−1^ molecular clock [62], Bayesian Skyline Plots revealed the assessment of the population fluctuation timing, which indicates relatively stable population size with a non-significant expansion trend during the LGM followed by a contraction (no more than three times) (Fig. 3).

### Present and LGM Distribution Areas

**Figure 4:**
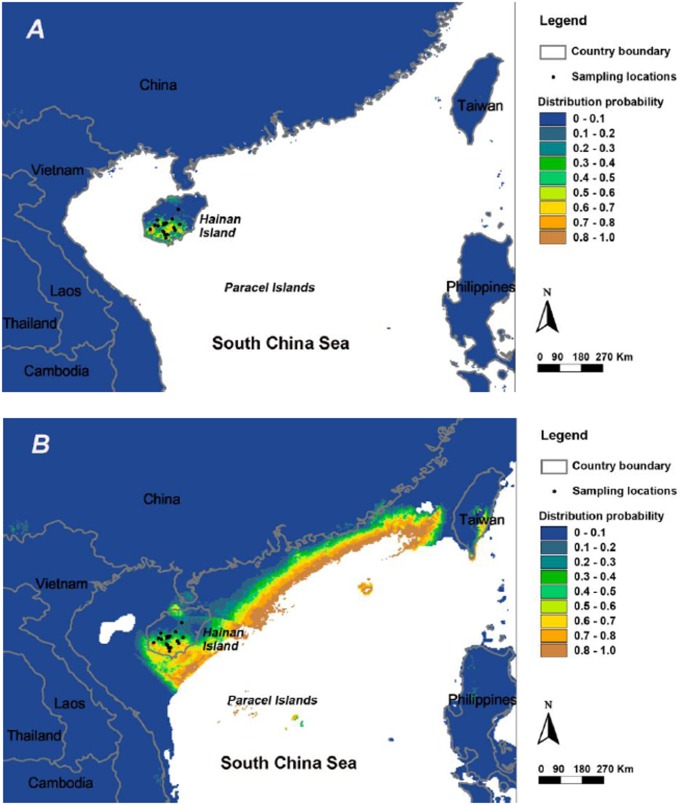
Map of the predicted distribution of *A. ardens* under current (A) and last glacial maximum (B). Distributions are shown from 0 (blue) and 1 (brown) probability. Note that the continental margin depicted on the LGM map differs from the other because sea level was lower during glacial periods.

For *A. ardens*, we modeled extant potential climatic distribution areas (Fig. 4A) based on the occurrence points of the species and projected it to the LGM (Fig. 4B). Based on current locality data, the high area under the curve (AUC) value of 0.996 for the model suggested a good fit between the model and data. The model of the extant potential distribution areas was a relatively accurate representation of the species’ realized distribution areas (Fig. 4A). Our results showed that its current distribution areas mostly lie in the middle and southern areas of the Hainan Island, including all of the extant surveyed distribution areas. Then we projected the models onto Southeast Asia’s climate conditions during the LGM, and resulted in predictions of potentially suitable habitats during ice age cold cycles (AUC = 0.97) (Fig. 4B). Specifically, results indicated that the current distribution of *A. ardens* on Hainan Island was not pushed toward warmer southern areas during the LGM but survived *in situ* in their extant distribution area on Hainan Island (Fig. 4B). It was indicated that climatic conditions during the LGM did not cause the total distribution contraction when comparing with its current distribution on the island. In contrast, projections from the species distribution models indicated an eastward expansion of distribution areas for *A. ardens* outside of current Hainan Island areas during the LGM (Fig. 4B). More suitable distribution areas became available along the coast between Hainan Island and Taiwan Island with the retreat of the sea level, which implied the current distribution is partly representative of its past.

## Discussion

In the present study, we coupled genetics analyses with SDMs to examine the responses of demographic history and distribution range of *A. ardens* on Hainan Island to the late Pleistocene climate. The results contradicted the predictions that populations contracted during the last ice age followed by a warming period expansion [4], but were consistent with the expectation that current range and demography were representative of their past during the LGM [7], [14]. In addition, a relatively long-term *in situ* survival of *A. ardens* on Hainan Island was indicated, which may be a distinct pattern of evolutionary history for upland species on Hainan Island.

### Molecular Demographic History and Genetic Structure

For *A. ardens*, the star-like network, values of statistical tests (non-significantly negative) and mismatch distributions (multimodal with significant values of goodness-of-fit tests) were not consistent with each other well. Bayesian skyline plots indicate a relatively constant population size, with a slight trend of past population expansion and recent slight decrease. Although BSP confidence intervals were large, the stability of populations is supported by the SDMs. Therefore, relatively stable historical demographic history could be deduced from our molecular information of mtDNA. SDMs indicate that the amount of suitable habitat available was constant for *A. ardens* on Hainan Island, during both the present and LGM, and a dramatic increased distribution outside of Hainan Island during LGM. Postglacial expansion events from potential refugia by climate warming were not detected in this study. This pattern was similar to forest community results that species in Southeast Asia once have survived ice ages with relatively stable demographic history during the LGM [7], [14], [63]. Some previous studies which focused on wildlife in Hainan Island supported the similar results of stable demographic history after postglacial periods [64]. Therefore, the lack of evidence of effective population size contraction during the LGM implied that *A. ardens* might have experienced local adaptation and coped with the glacial climate changes.

The detected weak genetic differentiation of sampling regions could be attributed to not only the connected distributions of *A. ardens* without geographical isolation, but also the potential gene exchange caused by seasonal altitude shifts for foods and mating as described by [57]. For example, fieldwork has once detected the overlapped home ranges owned by different individuals at varied altitudes in one region [57]. Similarly, evidence of weak intraspecific genetic divergence of species endemic to Hainan Island has also been detected and supported our results [28], [65], [66].

Our results and their interpretations are primarily dependent on the detected information of mtDNA for it can yield useful information about the historical processes behind intraspecific-level phylogeographic patterns, and its overall mutation rates tend to be higher than nuclear loci [67]. It is generally accepted that the mitochondrial hypervariable region (HVR-1 or the control region) evolved more rapidly than other mtDNA genes [68]. However, in the work about the phylogenetic relationships of the species in genus *Arborophila*, we did not detect more variable sites of HVR-1/CR than those of the cyt *b* and ND2 genes used in this study. Specifically, our previous work on the molecular diversity of Common Pheasant (*Phasianus colchicus*) in China also supported the lower diversity of HVR-1/CR than cyt *b* [69]. Even though the largest-ever samplings of Hainan Partridge are used, our ability to infer what was surely a complex demographic history is limited by the low mtDNA genetic variations. To reveal detailed processes in contemporary landscape genetics and demographic history of Hainan Partridge influenced by recent anthropogenic activities, there is a need to pursue additional highly variable nuclear markers such as SNPs (single nucleotide polymorphisms) or microsatellites DNA. However, these first combined data for Hainan Partridge demonstrate the value of paired genetic and SDMs studies. Moreover, they provide an excellent framework that, with the addition of molecular markers (such as SNPs or microsatellites DNA) or endemics species on Hainan Island [70], may become an important piece in the tropical biodiversity and evolutionary debates.

### Present and LGM Distribution Areas

We detected the pattern of more suitable distribution areas becoming available along the coast between Hainan Island and Taiwan Island with the retreat of the sea level, which implied the current distribution is partly representative of its past. Therefore, the current distribution of *A. ardens* on Hainan Island could be recognized as *in situ* refuge. That is, the total distribution of *A. ardens* before and during the LGM had once been located on the middle and southern areas of Hainan Island as the responses to the late Quaternary climate. Although the paleodistribution models of *A. ardens* supported the hypothesis of their *in situ* survival of the glaciations periods on Hainan Island, the extent of genetic diversity and demographic dynamics of *A. ardens* during the LGM did not coordinate well with the expansion of the modeled LGM distribution areas.

There are four possible reasons for this: 1) because soils are particularly important in structuring tropical forest communities, the repeated submersion and exposure of soils on the shelf may have affected their structure and fertility significantly [71]. The evolution of these soils, after exposure, and their interaction with advancing rainforests would have been important, although nothing is known about these dynamics. During the LGP, it has been hypothesized that rainforest was partly replaced by savanna over large areas in Southeast Asia [20]. However, *A. ardens* has never been found in the savanna habitats; 2) because of the repeated sea-level changes. It seems conceivable that potential distribution on coastal areas and populations on those during the LGM might have vanished by gradually rising sea levels. A recent study showed there were >58 rapid sea-level rises of >40 m in the last 5 Myr in Southeast Asia [72], which would have resulted in significant habitat fluctuation and local population extirpation in the narrow coastal parts between Hainan Island and Taiwan Island; 3) *A. ardens* was more susceptible to cold temperature during the LGM. Field studies have demonstrated that this pheasant was sensitive to cold temperature. Some adults of *A. ardens* could freeze to death in winter [57]. In addition, the lower seasonal dispersal ability of this pheasant during the colder period, such as winter, has been described [40], [57]. Although the potential distribution areas increased during the LGM, populations of the pheasant might not expand accordingly; and 4) although the demographic and distribution patterns described here are all historical events, very recent human-induced activities might affect the range of variation of extant genetic information.

It should be noted, however, that the paleodistribution was calculated for the LGM (about 21,000 years ago) only and not the entire ice age, which might have differed from the LGM at certain times during the Pleistocene. Therefore, our models just showed the possibility of the occurrence of *A. ardens* on Hainan Island during glaciation cycles. They cannot exclude that extended land areas or more severe climate conditions might have influenced the divergence and demography of *A. ardens* populations earlier. Furthermore, such models do not take into account the potential effects of biotic exclusion, dispersal limitation, or historical contingency on species ranges. As such, it is important to recognize that these models reflect species potential ranges rather than their realized ranges. Transferable modeling involves projecting SDMs onto different geographic areas or different time periods and has been used for predicting species responses to global warming, reconstructing paleo-ranges of organisms, or modeling the spread of invasive species (e.g. [35], [73]). Although this approach is still challenging due to conceptual assumptions (e.g. niche conservatism through time) as well as uncertainties (e.g. extrapolation) associated with the methodology [74], we believe these difficulties can be offset by the potential benefits of improving studies of the population processes that contribute to regional patterns of biodiversity by connecting micro-evolutionary processes to macro-evolutionary patterns. In addition, higher-resolution paleo-environmental data sets such as the LGM climate data used herein are increasingly available, so further related research in this realm should be increasingly fruitful. Moreover, finer-scale and biologically-relevant paleo-environmental layers (e.g., soil types, land cover) will likely increase further the quality and resolution of SDMs predictions.

### Conservation Implications

According to the relatively stable historical effective population size and stable distribution areas on Hainan Island through the LGM, *A. ardens* might have formed local adaptations during those times and survived the late Quaternary glaciations. During the past decades, the fragmented habitat and small population size of extant *A. ardens* have been attributed to human-induced activities such as over-logging and poaching. Fortunately, the discovery of a number of new populations since 2002 and the subsequent protection of more forest where the species occurs suggests that this rate of decline may have slowed or even ceased in recent years [43], [44]. Small populations are generally considered to be susceptible to a number of genetic problems like low level of variability, inbreeding depression, and the ability to overcome disease agents. Therefore, we recommend that conservation efforts be made to avoid the anthropogenic population decline for the *A. ardens* on Hainan Island. Moreover, due to the weak population genetic structure and current *in situ* refuge stage on Hainan Island, we suggest that extant populations of *A. ardens* on Hainan Island should be considered as one management unit.

## Supporting Information

Table S1 Description of 19 bioclimatic variables used in ecological niche modeling. Variables are derived from the monthly temperature and rainfall values in order to generate more biologically meaningful variables. The bioclimatic variables represent annual trends (e.g., mean annual temperature, annual precipitation), seasonality (e.g., annual range in temperature and precipitation), and extreme or limiting environmental factors (e.g., temperature of the coldest and warmest month, and precipitation of the wet and dry quarters).
(DOC)
