# Supplementary material for: Coupling Genetic and Species Distribution Models to Examine the Response of the Hainan Partridge (Arborophila ardens) to Late Quaternary Climate
Source: PLoS One. 2012 Nov 19;7(11):e50286. doi: 10.1371/journal.pone.0050286 (PMC3501459; doi:10.1371/journal.pone.0050286)
Supplement: Table S1 — Description of 19 bioclimatic variables used in ecological niche modeling. Variables are derived from the monthly temperature and rainfall values in order to generate more biologically meaningful variables. The bioclimatic variables represent annual trends (e.g., mean annual temperature, annual precipitation), seasonality (e.g., annual range in temperature and precipitation), and extreme or limiting environmental factors (e.g., temperature of the coldest and warmest month, and precipitation of the wet and dry quarters). (DOC) [file pone.0050286.s001.doc]

**Supporting information**

**Table S1** Description of 19 bioclimatic variables used in ecological niche modeling. Variables are derived from the monthly temperature and rainfall values in order to generate more biologically meaningful variables. The bioclimatic variables represent annual trends (e.g., mean annual temperature, annual precipitation), seasonality (e.g., annual range in temperature and precipitation), and extreme or limiting environmental factors (e.g., temperature of the coldest and warmest month, and precipitation of the wet and dry quarters).

| Variable code | Description |
| --- | --- |
| BIO1 | Annual Mean Temperature |
| BIO2 | Mean Diurnal Range (Mean of monthly (max temp - min temp)) |
| BIO3 | Isothermality (BIO2/BIO7) (* 100) |
| BIO4 | Temperature Seasonality (standard deviation *100) |
| BIO5 | Max Temperature of Warmest Month |
| BIO6 | Min Temperature of Coldest Month |
| BIO7 | Temperature Annual Range (BIO5-BIO6) |
| BIO8 | Mean Temperature of Wettest Quarter |
| BIO9 | Mean Temperature of Driest Quarter |
| BIO10 | Mean Temperature of Warmest Quarter |
| BIO11 | Mean Temperature of Coldest Quarter |
| BIO12 | Annual Precipitation |
| BIO13 | Precipitation of Wettest Month |
| BIO14 | Precipitation of Driest Month |
| BIO15 | Precipitation Seasonality (Coefficient of Variation) |
| BIO16 | Precipitation of Wettest Quarter |
| BIO17 | Precipitation of Driest Quarter |
| BIO18 | Precipitation of Warmest Quarter |
| BIO19 | Precipitation of Coldest Quarter |
